# Local Adaptation? Enhanced Fitness Under Regional UVB Intensities in a Rock Pool Bdelloid Rotifer

**DOI:** 10.1002/ece3.72256

**Published:** 2025-10-28

**Authors:** Maribel J. Baeza, Elizabeth J. Walsh

**Affiliations:** ^1^ Department of Biological Sciences University of Texas at El Paso El Paso Texas USA

**Keywords:** Chihuahuan Desert, ephemeral habitats, lifetable experiments, maternal effects, phenotypic plasticity, ultraviolet radiation

## Abstract

Exposure to ultraviolet radiation (UVR) can decrease lifespan and reduce fecundity in aquatic invertebrates. Organisms inhabiting shallow waters are often unable to avoid UVR damage. Bdelloid rotifers are known for their resistance to extreme environments and ionizing radiation. However, conflicting results have been reported regarding their response to UVR. We hypothesized that exposure to UVB radiation would negatively affect survival and other life history characteristics (i.e., lifespan, net reproductive rate, intrinsic rate of increase) with increasing intensities and across multiple generations in bdelloids that inhabit shallow rock pools. To test these hypotheses, field‐collected females (F_0_) were exposed to two environmentally relevant and one extreme level of UVB for 2 h and individuals that survived were retained for further analysis. Their F_1_ neonates were then cultured, and their offspring (F_2_, F_4_) were exposed to the same UVR treatment as the parental generation, and survival was again recorded. Lifetable analyzes were conducted using offspring of exposed mothers in the F_1_ and F_5_ generations. As predicted, exposure to UVB radiation negatively affected survival and resulted in cumulative damage after each subsequent exposure to UVB at all intensities. However, maternal exposure to low UVB levels resulted in increased lifespan (97%) and net reproductive rate (215%) in their progeny. At mid UVB intensities, net reproductive rate increased but to a lesser extent. This may reflect an adaptive response to environmental stress, enabling faster reproduction. However, increasing UVR intensity has adverse effects on longevity and fecundity which may compromise population fitness.

## Graphical abstract

Bdelloids from the Chihuahuan Desert appear to have adapted to regional UVB intensities in a localized manner. Bdelloids exposed to winter or summer levels of UVB intensity had a longer lifespan and a higher net reproductive rate.

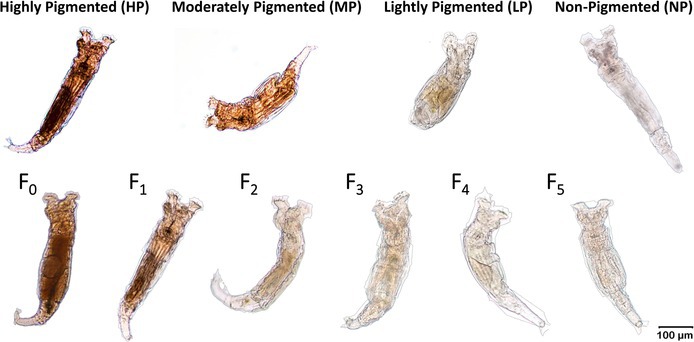

## 1 Introduction

The ability of animals to respond to global climate change will depend on their capacity to adapt to environmental stressors. Climate change is predicted to affect weather patterns, prolong droughts, increase temperatures, and directly and indirectly intensify ultraviolet radiation (UVR) (Bais et al. 2018; Chatzopoulou et al. 2025; Salawitch et al. 2018). Directly, climate change increases UVR intensity by contributing to ozone depletion. Indirectly, changes in weather patterns can decrease cloud coverage, leading to longer periods of UVR exposure (Bais et al. 2018; Chatzopoulou et al. 2025; Salawitch et al. 2018). The intensity of UVR in any environment fluctuates naturally based on the day, season, latitude, and elevation (Chatzopoulou et al. 2025; Häder et al. 2007; McKenzie et al. 2011). During the summer, UVR intensity increases by 8%–10% every 1000 m above sea level (Blumthaler et al. 1997; Piazena 1996; Williamson et al. 2001). This makes high altitude areas of the Chihuahuan Desert (> 1000 m) more prone to UVR exposure (Baeza and Walsh 2024). Thus UVR exposure may be acting as a selective force favoring individuals who can mitigate or prevent damage (Baeza and Walsh 2024; Fernández et al. 2018; Häder et al. 2007; Marinone et al. 2006), especially for those inhabiting aquatic systems.

Maternal effects can further enhance adaptation by influencing offspring traits in response to environmental challenges (Dam 2013; de Villemereuil et al. 2022; Fox et al. 2019; White and Butlin 2021). Life table experiments have been instrumental in studying such adaptations. For example, the cladoceran *Daphnia pulex* Leydig, 1860 evolved tolerance to salinity with associated trade‐offs in reproductive output, lifespan, and overall variability in fitness—tolerant individuals produced fewer offspring, which had shorter lifespans and more variability in fitness across generations (Hinz et al. 2018). Similarly, the copepod *Pseudodiaptomus annandalei* Sewell, 1919 exhibited adaptive maternal effects under copper stress (Dinh et al. 2020). *Daphnia magna* (Straus, 1820) displayed age and size‐dependent heat tolerance, where larger individuals showed greater resistance but at the cost of delayed reproduction (Burton et al. 2020). In the rotifer *Brachionus dorcas* Gosse, 1851, Ge et al. (2025) found differences in lifespan, reproduction, and morphology across spine morphotypes in response to thermal stress; individuals with short spines exhibited enhanced developmental plasticity and survival under colder conditions. Other studies have demonstrated maternal effects of phosphorus limitation on offspring performance in *Brachionus calyciflorus* Pallas, 1766 (Zhou and Declerck 2020) and salinity tolerance thresholds of *Moina macrocopa* (Straus, 1820) (Yuslan et al. 2021). Phenotypic responses to changes in salinity, nutrient limitation, or thermal stress emphasize the potential for environmental stressors to drive divergent evolution.

In addition to environmental conditions, multi‐generational exposure to stressors can shape adaptive outcomes, though with inconsistent effects across species. For instance, populations of *D. pulex* from high UVR environments showed increased fecundity and earlier reproduction, while populations from low UVR regions had shorter lifespans and lower fecundity (Fernández et al. 2018). Further, in the low UVR populations, photoprotective pigments were produced which help to mitigate UVR damage, while in high UVR populations (which were pigmented), individuals moved to refuge in deeper regions of the lake (Fernández et al. 2020). Similarly, populations of *Daphnia ambigua* Scourfield, 1947 from a clear lake accumulated carotenoids and melanin, while the same species from a lake with more turbid water lacked pigmentation altogether. *Leptodiaptomus cuauhtemoci* (Osorio‐Tafall, 1941), which was only found in the high‐UVR lake, accumulated mycosporine‐like amino acids (MAAs; small, UV absorbing compounds) and carotenoids as protective compounds (Alcocer et al. 2020). Other copepods (i.e., *Cyclops abyssorum* G.O., 1863, *C. abyssorum tatricus* Koźmiński, 1927, and *Acanthodiaptomus denticornis* (Wierzejski, 1887)) showed elevated MAA concentrations in lakes with high UV transparency (Leech and Williamson 2001). This variability in pigmentation and photoprotective compound production underscores their importance in UVR tolerance, providing a significant survival advantage in high‐UVR habitats, particularly in areas with few visual predators (e.g., Alcocer et al. 2020; Hansson 2004; Hylander et al. 2009).

Species‐specific differences in UVR adaptations are evident, with some zooplankton demonstrating photoprotective mechanisms, while others show a trade‐off or delayed responses to UV exposure. In *D. magna* , no adaptation was observed in F_2_ offspring after the previous two generations had been exposed to UVB radiation, indicating a lack of immediate adaptive capacity (Sha et al. 2020). Although clones from low‐UVR habitats exhibited earlier reproduction and higher fecundity, suggesting a more pronounced response in populations from lower UVR environments (Sha et al. 2020). In the copepod *Tigriopus californicus* (Baker, 1912), exposure to moderate UVB/A intensities enhanced fecundity, which led to larger clutch sizes (Heine et al. 2019). A similar pattern was observed in *Brachionus urceus* (Linnaeus, 1758), where lower UVB doses resulted in increased fecundity (Wang et al. 2011). These reproductive benefits may ensure the survival of the species under UVB stress at the cost of reduced longevity and survival of the individual (e.g., Heine et al. 2019; Sha et al. 2020; Wang et al. 2011).

Shallow ephemeral rock pools at high elevations are particularly vulnerable to climate change, with increased UVR, rising temperatures, and prolonged droughts (Schröder et al. 2007; Walsh et al. 2014). Bdelloid rotifers that are common in these habitats can endure extreme stressors, including desiccation (Caprioli and Ricci 2001; Hespeels et al. 2014, 2023; Hinz et al. 2018), freezing (Shain et al. 2024), starvation (Marotta et al. 2012), and ionizing radiation (Gladyshev and Meselson 2008; Hespeels et al. 2014, 2023). Bdelloids achieve this resilience by entering dormancy as a xerosome (Wallace et al. 2008). However, despite their adaptability, some bdelloids remain vulnerable to UVR‐induced declines in reproductive output, longevity, and overall fitness (Fischer et al. 2013; Zhu et al. 2021). For example, *Rotaria rotatoria* (Pallas, 1776) exhibited delayed reproduction, reduced fecundity, and shortened longevity under increasing UVB intensity (280–312 nm) (Zhu et al. 2021). Similarly, *Philodina roseola* Ehrenberg, 1832, produced fewer eggs when exposed to UVB radiation (Fischer et al. 2013; Hinz et al. 2018). These species employed various biochemical defenses such as antioxidant enzymes, but these mechanisms did not prevent the negative impacts UVB had on survival or reproduction (Fischer et al. 2013; Zhu et al. 2021).

The role of pigmentation in mitigating UVR stress remains understudied in rotifers, though Baeza and Walsh (2024) demonstrated that pigmentation level directly influenced the survival of a rock pool bdelloid. They found that the level of pigmentation played a direct role in UVB protection, with more pigmented individuals demonstrating a higher resistance to UVB radiation. Similarly, small, pigmented rotifers such as *Keratella taurocephala* Myers 1938, *Keratella cochlearis* (Gosse, 1851) and *Polyarthra dolichoptera* Idelson, 1925, all known to have MAAs, exhibited consistently high UVR tolerance (Leech & Williamson 2000; Obertegger et al. 2008; Tartarotti et al. 2001). This was supported by field observations of these species occurring near the surface of clear lakes as opposed to the larger, more transparent *Asplanchna priodonta* Gosse, 1850, which was found deeper in the lake where UVR could not penetrate (Leech and Williamson 2001; Obertegger et al. 2008). These physiological adaptations demonstrate how long‐term exposure to UVR can influence species‐specific survival strategies in clearwater ecosystems and underscore the ecological significance of UVR as a driver of functional diversity within freshwater zooplankton communities (Leech and Williamson 2001; Obertegger et al. 2008).

To investigate the effects of UVB exposure on life history traits over multiple generations, we used the same study system as Baeza and Walsh (2024). We hypothesized that life history traits, such as lifespan, fecundity, and reproduction of a rock pool bdelloid would be negatively affected by exposure to increasing UVB intensities. Furthermore, we predicted that UVB exposure over multiple generations would have cumulative adverse effects, negatively impacting life history traits in subsequent generations. To test these hypotheses, we exposed the bdelloid to three levels of UVR over three generations and conducted life table experiments on surviving offspring. Specifically, we determined the survival of individuals from the parental (F_0_), F_2_, and F_4_ generations and assessed life history traits of their offspring (F_1_ and F_5_ generation).

## 2 Methods

### 2.1 Collection and Culture

The parental generation (F_0_) of an undescribed *Philodina* species (hereafter *Philodina*) was collected from rock pools at Hueco Tanks State Park and Historic Site, El Paso Co., TX (hereafter Hueco Tanks). A complete description of the study site is given in Baeza and Walsh (2024). Water samples were collected 48–72 h after a significant rain event (≳ 0.5 cm). To account for acclimation to environmental UVB intensities, rotifers were exposed to UVB treatments according to the season they were collected. Individuals for the control and low UVB treatments were collected in the winter (February 2020, January 2021) when regional mean UVB intensities were ~1.4 W/m^2^ (Sengupta et al. 2018). During this time, temperatures ranged between 1°C and 20°C (retrieved December 15, 2022, https://www.weather.gov/epz/climatedataforelpaso). Specimens for the mid and high UVB treatments were collected in late summer (September 2020 and September 2019 respectively) when regional mean UVB intensity was ~3.5 W/m^2^, accompanied by high temperatures (~35°C–40°C). In the laboratory, rotifers were washed free of debris using modified MBL media (Stemberger 1981) and then fed the green alga *Chlamydomonas reinhardtii* (Dangeard, 1888; Culture Collection of Algae at the University of Texas at Austin strain 90). Cultures were maintained in an 18:6 h L:D cycle at 25°C ± 1°C.

### 2.2 Generational Exposure to UVR

Three non‐consecutive generations of *Philodina* were exposed to two environmentally relevant levels of UVB, as well as an extreme case scenario. The F_0_ generation was exposed within 72 h of collection, while F_2_ and F_4_ were exposed at the end of their juvenile period (10–14 days old) (Caprioli and Ricci 2001; Ricci 1998; Ricci and Covino 2005). Pigmentation levels were determined based on analysis of 25 xerosome images following Baeza and Walsh (2024). Briefly, pigment levels were determined based on %red digital number (DN); highly pigmented (HP: > 45%), moderately (MP: 38%–42%), lightly pigmented (LP: 36%–37.4%), and non‐pigmented (NP: < 36).

Rotifers (16 replicates; *n* = 50) were exposed to UVB radiation in 2 mL of MBL and at UVB intensities of: low (1.3 ± 5 W/m^2^), mid (3.7 ± 5 W/m^2^), or high (5.0 ± 5 W/m^2^) for 2 h at 25°C± 1°C. UVB radiation was emitted from a Spectroline XX‐15B UVR lamp with two UVB bulbs (280–400 nm, peak output at 306 nm; Ushio G15T8E Hammond, IN). The lamp was suspended above the Petri dishes in a low‐temperature, diurnal illumination incubator (VWR Model 2015). A glass filter was used for the low UVB treatment so that only wavelengths ≥ 305 nm reached the Petri dish. A quartz glass filter resulted in rotifers being exposed to the full spectrum emitted by the UVB lamp (280–400 nm) for the mid and high UVB treatments. Intensities were verified using a UVA/B light meter (Sper Scientific 850,009). Negative controls were treated the same but placed in a styrofoam box covered in black plastic during exposures. Additional experimental setup details are given in Baeza and Walsh (2024). After 48 h, individuals that showed visible trophi (jaw) movements were considered alive. Livining individuals (F_0_, F_2_, and F_4_) were cultured with algal food as above, neonates produced within a week of UVB exposure were moved to a separate Petri dish with algal food to generate F_1_, F_3_, and F_5_ offspring, respectively. Offspring of the F_1_ or F_5_ generation that hatched within a 6 h period were selected for lifetable experiments.

### 2.3 Life History Characteristics

Life table experiments were done in replicates of 3 (F_1_: control, mid, high UVB exposure; F_5_: control) or 5 (F_1_: low; F_5_: low and mid UVB exposure) in a 9‐well glass plate with one female per well. Rotifers were fed 250,000 ± 25,000 cells/mL of *C. reinhardtii* and cultured under an 18:6 h L:D cycle at 25°C± 1°C. Algae were counted daily, diluted to the appropriate concentration before removing old food from each well and replacing it with 850 μL of fresh food. An additional set of 3 replicates per UVR treatment was also followed but starved, serving as a control since the nutritional state influences life history traits and physiological resilience in rotifers (Kirk 2012; Marotta et al. 2012). The number of females alive and neonates that hatched was recorded every 24 h. Females were moved to a new 9‐well plate weekly to prevent the overgrowth of algae and accumulation of bacteria while eggs were retained until neonates hatched. Neonates were then moved to a new plate with fresh media with food for further experimentation. The number of surviving females and neonates hatched was used to calculate age‐specific survivorship (*l*_*x*_), fecundity (*m*_*x*_), the net reproductive rate (*R*_0_), generation time (*T*), and the intrinsic rate of population increase (*r*) using Equations ((1), (2), (3), (4), (5)), respectively:

(1) $$l_{x}\;=\;\frac{n_{x}}{n_{o}}$$

(2) $$m_{x}=\frac{b_{x}}{n_{x}}$$

(3) $$R_{0}=\sum l_{x}m_{x}$$

(4) $$T=\sum \frac{{\mathrm{xl}}_{x}m_{x}}{R_{0}}$$

(5) $$\;r\sim \frac{\ln\left(R_{0}\right)}{T}$$

where *x* is time (day), *n*_0_ the starting number of females, *n*_x_ is the number of remaining survivors each day, and *b*_x_ is the number of neonates (Caswell 1989). In this study neither *l*_*x*_ nor *m*_*x*_ was analyzed but they were used to calculate *R*_0_, *T*, and *r*.

## 3 Statistics

### 3.1 Generational Exposure to UVR

Survival, measured as the number of living rotifers after treatment, was determined in the F_0_, F_2_, and F_4_ generations. A Shapiro–Wilk test was used to determine whether the data were normally distributed, and a Levene's test was used to examine homogeneity of variance. A two‐way analysis of variance (ANOVA) was conducted to determine the significance of UVB treatment, generation, and their interaction on rotifer survival. When significant differences were detected, a Tukey post hoc test was conducted to determine pairwise differences between UVB treatment groups and generations. Statistical analyzes were performed using R version 3.4.3 (R Core Team 2022) and RStudio version 1.0.136 (RStudio Team 2020).

### 3.2 Life History Characteristics

Survivorship and other life history parameters were analyzed to assess the effects of maternal UVB exposure on offspring fitness. Multivariate Cox proportional hazards (PH) regression survival analyzes (Kragh Andersen et al. 2021) were used to evaluate the impact of increasing UVB radiation and single or multiple maternal UVB exposures on lifespan and generation time (*T*). Cox PH models were fitted using the Survival package (Therneau 2022), and mixed‐effects Cox models were implemented with the coxme package (Therneau and Grambsch 2000a, 2000b). The Kenward–Roger correction was applied to the model to adjust the degrees of freedom to account for variance in the data and minimize type I errors (Kenward and Roger 2009).

Net reproductive rates (*R*_0_) and intrinsic rate of increase (*r*) were analyzed using a linear mixed effects (lme) model. Random effects were included in the model to account for variation across replicates and to isolate the effect of UVB treatment on bdelloid survival. These analyzes were performed using the lme4 package (Bates et al. 2015). Pairwise comparisons between UVB treatments as well as comparing the F_5_ to the F_1_ generations for each model were performed using estimated marginal means with the emmeans package (Lenth 2023) and the multcomp package (Hothorn et al. 2008). All statistical analyzes were conducted in R (version 3.4.3; R Core Team 2022) and RStudio (version 1.0.136).

## 4 Results

### 4.1 Generational UVR Exposure

**FIGURE 1:**
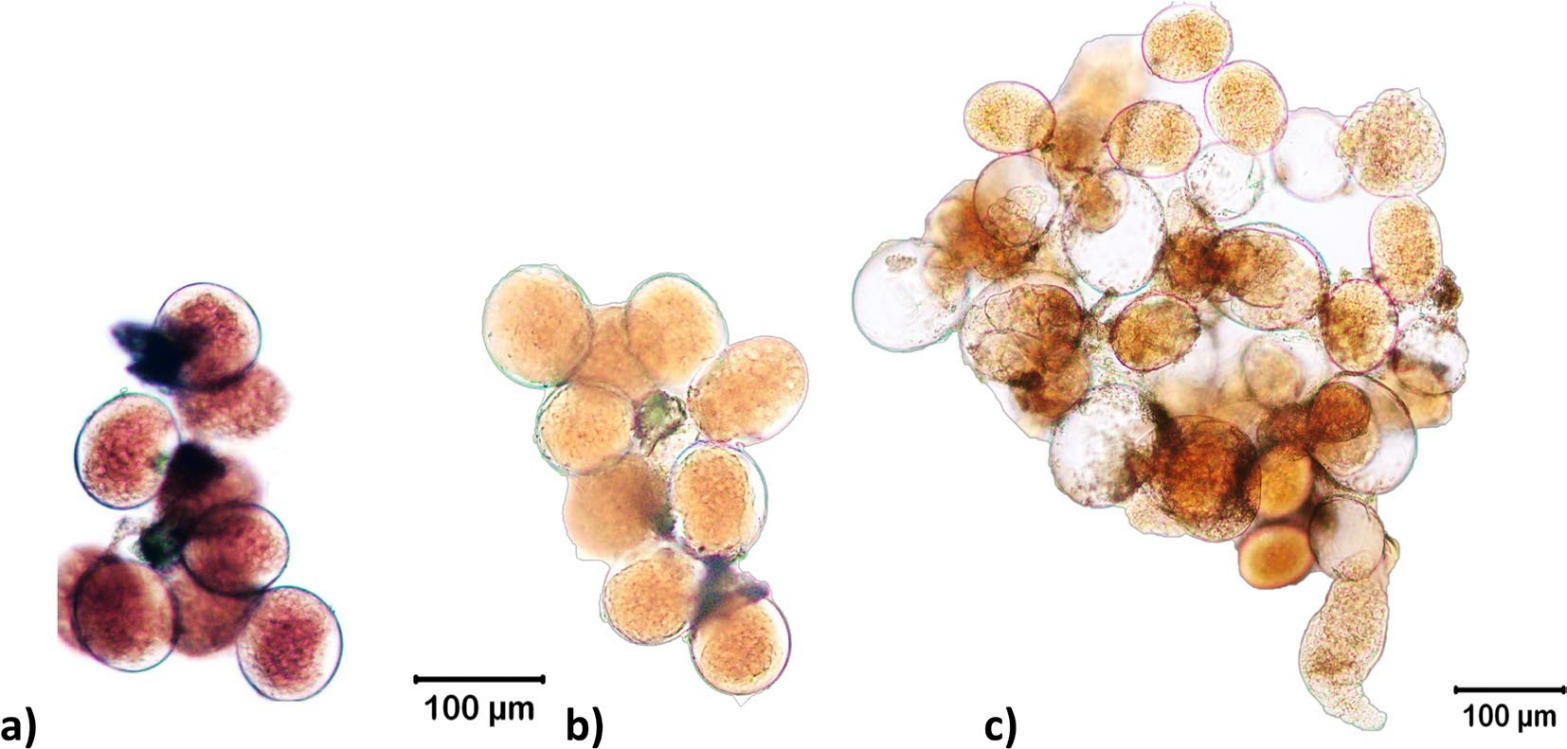
*Philodina* embryos: (a) Pigmented embryos produced by F_0_ generation *Philodina* females, (b) F_1_ embryos after 1 week of laboratory culture, (c) embryos after 2 weeks of laboratory culture, pigmented neonates, and clear eggshells. (a) and (b) share a common scale bar.

**FIGURE 2:**
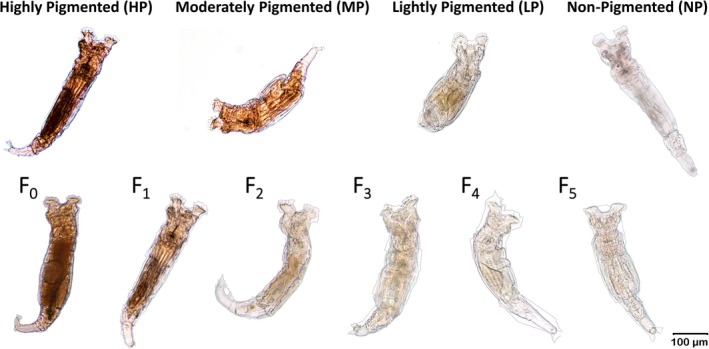
Unexposed *Philodina* showing (upper series) pigmentation categories and (lower series) changing levels of pigmentation over generations.

The parental generation of *Philodina* (F_0_) had a red digital number of 45.8% ± 0.7%, corresponding to highly pigmented (Tables 1 and S1). Eggs laid by the parental generation were also highly pigmented (HP; Figure 1a); this resulted in pigmented F_1_ offspring (Figure 2). The pigmented F_1_ adults laid slightly less pigmented eggs (Figure 1b) and neonates. The F_2_ offspring hatched and left behind a clear eggshell (Figure 1c). Analysis of pigmentation in F_2_ adult xerosomes resulted in 40.36 ± 1.1% red pixels (Tables 1 and S1), indicating a moderate pigmentation level (Figure 2). In F_4_ offspring, red pigmentation was further reduced to 36.2% ± 0.3% red pixels (Figure 2). Despite this decline, slight red coloration remained in the intestinal lining; thus, F_4_ xerosomes were between pigmentation levels of lightly pigmented and non‐pigmented (Tables 1 and S1).

**TABLE 1:** Pigmentation levels in *Philodina* xerosomes over generations before exposure to UVB.

Generation	Mean ± SD total DN	Mean ± SD Red DN	% Red DN	Pigment level assignment
F_0_	308 ± 35	141 ± 22	45.8^a^	HP
F_2_	450 ± 23	181 ± 24	40.4^b^	MP
F_4_	624 ± 34	226 ± 9	36.2^c^	LP/NP

*Note:* Images of xerosomes were captured and analyzed by counting the number of pixels in the red channel of each image. *n* = 25, mean ± standard deviation (SD). All comparisons were significant at *p* < 0.05; see Table S1. Superscript letters indicate a significant difference in the % red digitial number (DN) see Table S1.

Abbreviations: HP, highly pigmented; MP, moderately pigmented; LP, lightly pigmented; NP, non‐pigmented.

**FIGURE 3:**
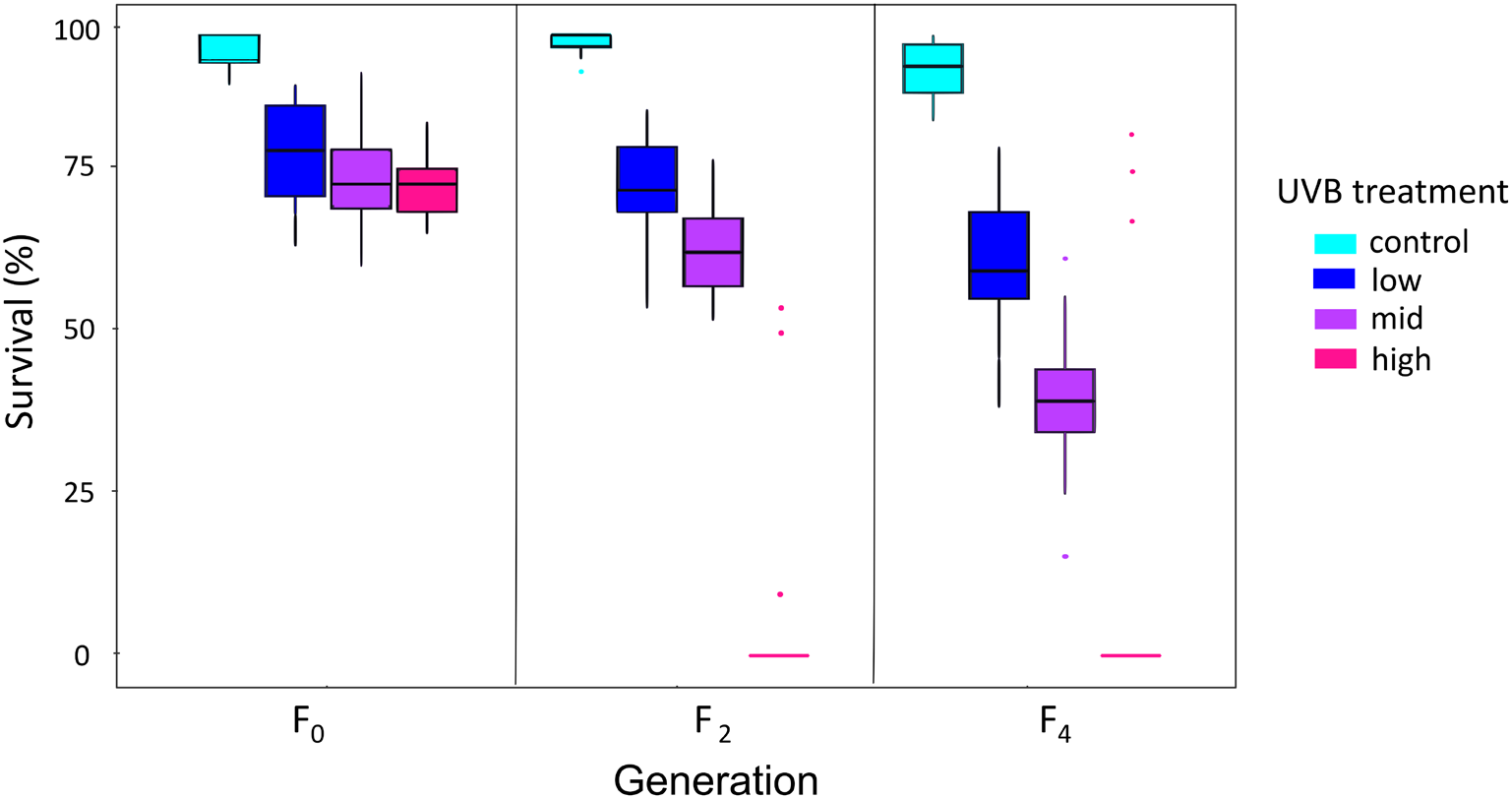
Effects of three levels of UVB exposure (1.3, 3.7, 5.0 W/m^2^) on survival of *Philodina* over three generations (F_0_, F_2_, and F_4_ offspring). In the box plots, the horizontal bar represents the median, the top represents the third quartile, and the bottom represents the first quartile. Dots indicate outliers. Significant different results are given in Table S2.

The number of alive individuals in unexposed treatments in the F_0_, F_2_, and F_4_ generations were 69%–17% higher than in UVB exposed treatments; thus, the decrease in survival can be attributed to UVB exposure (Figure 3). Negative effects of UVB intensity became more apparent in the F_4_ generation with survival decreasing by 22%, 50%, and 59% for bdelloids in the low, mid, and high UVB treatments, respectively (ANOVA, *F* = 29.6, df = 6, *p* < 0.0001; Tables 2 and S2). Bdelloid survival also significantly decreased when comparing the F_4_ to the F_0_ generation (Figure 3 and Table S2). Survival decreased to 14% in F_2_ and 7% in F_4_ generation offspring, respectively (Figure 3 and Table S2). The number of viable offspring produced by the F_4_ generation when exposed to high UVB, was 21, which was insufficient to conduct F_5_ lifetable experiments.

**TABLE 2:** The effects of UVB exposure on survival across three non‐consecutive generations of *Philodina* (F_0_, F_2_, and F_4_) were analyzed using a two‐way ANOVA.

	df	Sum of squares	Mean of squares	*F*	*p*
UVB intensity	3	10.28	3.43	208.9	< 0.0001
Generation	2	2.89	1.45	88.1	< 0.0001
UVB intensity: Generation	6	29.60	0.49	29.6	< 0.0001
Residuals	180	2.95	0.02		

*Note:* Tukey's Honest Significance Difference (HSD) results are given in Table S2.

Abbreviation: df, degrees of freedom.

### 4.2 Life History Characteristics

Lifespan analyzes were conducted using only fed treatments and excluding the high UVB treatment, while the full dataset was retained for other life history parameters. Including the starved and high UVB treatments resulted in quasi‐separation in the data due to the extreme values observed in both groups. A penalty was applied to the Cox PH model for lifespan.

In the F_1_ generation, both fed and starved individuals exhibited increased lifespans under low and mid UVB exposure compared to the control, but lifespan declined under high UVB. Fed F_1_ individuals had the longest lifespan under low UVB (69.6 ± 35.0 days; Table 3), with 4% surviving beyond 100 days. Starved F_1_ individuals also lived longest under low UVB (46.0 ± 7.8 days; Table 3) but had the shortest lifespan under high UVB (20.7 ± 6.2 days; Table 3).

**TABLE 3:** Lifespan (days, mean ± standard deviation) of two generations (F_1_ and F_5_) of *Philodina* after maternal exposure to low, mid, high, and control UVB radiation (0, 1.3, 3.7, or 5.0 W/m^2^).

	Control	Low	Mid	High
F_1_ fed	32.1 ± 9.8	69.6 ± 35.0	38.4 ± 6.4	21.5 ± 9.0
F_1_ starved	24.5 ± 5.1	46.0 ± 7.8	33.4 ± 1.0	20.7 ± 6.2
F_5_ fed	39.1 ± 11.1	58.7 ± 25.1	55.9 ± 21.7	ND
F_5_ starved	22.1 ± 2.5	40.5 ± 15.3	39.8 ± 4.3	ND
Mean F_1_ lifespan	28.3 ± 5.4	55.7 ± 14.1	35.9 ± 4.9	21.1 ± 4.5
Mean F_5_ lifespan	30.6 ± 10.2	49.6 ± 14.2	47.8 ± 10.3	ND

Abbreviation: ND, no data (due to low reproduction of F_4_ females).

**FIGURE 4:**
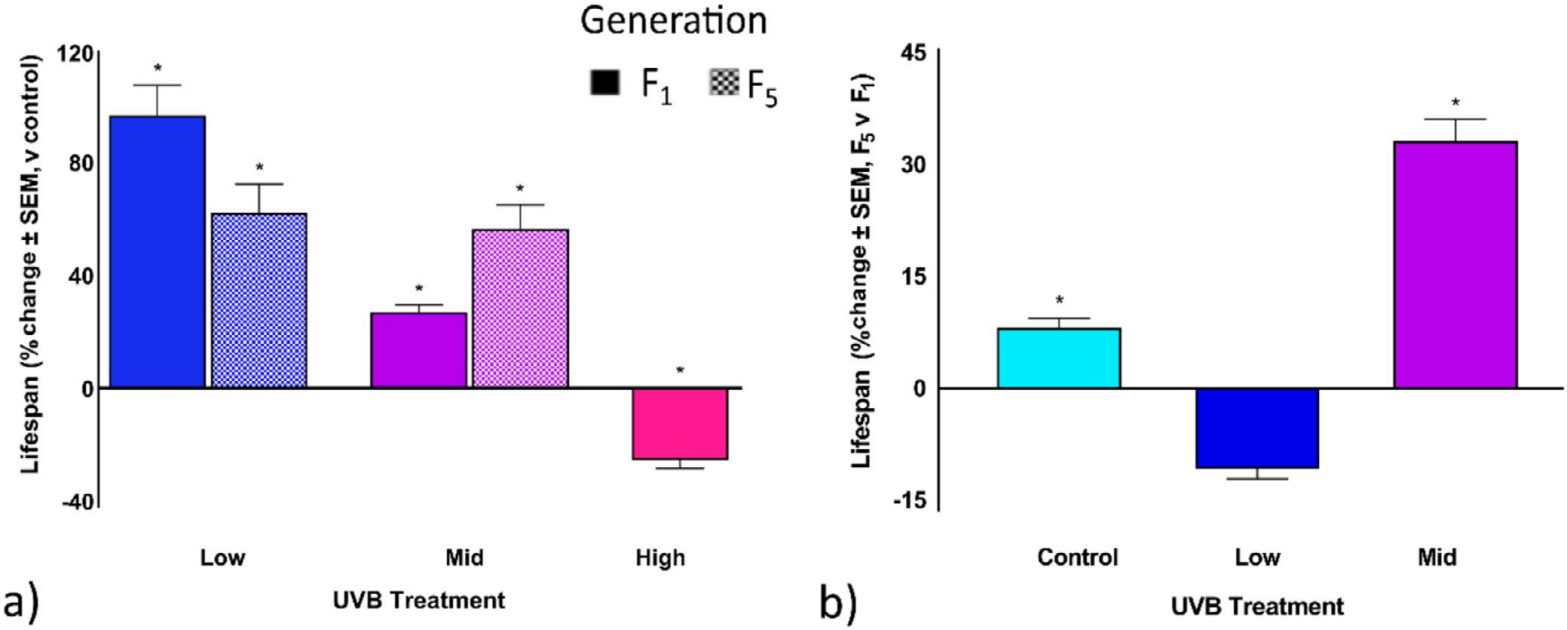
Maternal UVB exposure and transgenerational effects on *Philodina* lifespan. Bdelloids were exposed to three levels of UVB radiation (0, 1.3, 3.7, or 5.0 W/m^2^). Lifespans were (a) compared to the respective generation control and (b) compared for the F_5_ generation versus the F_1_ generation. *Estimated marginal means, *p* < 0.05. SEM, standard error of means.

To assess the effects of maternal UVB exposure, the lifespan of bdelloids from each UVB treatment was compared to the generation control treatment. Lifespan increased in the low and mid UVB treatments by 96.8% and by 26.9%, respectively (Figure 4, *p* < 0.0001; Table 4a), but decreased by 33.0% in the high treatment for the F_1_ generation (Figure 4a). In the F_5_ generation, lifespan increased by 62.1% in the low UVB treatment (Figure 4a; *p* < 0.0001; Table 4a), and by 56.2% in mid UVB cohorts (Figure 4a, *p* < 0.0001; Table 4a). When comparing the F_5_ generation to the F_1_, lifespan increased in control by 8.1%, by 28.6% in the mid UVB treatment (Figure 4b, *p* < 0.0001; Table 4b), and decreased by 31.0% in the low UVB treatment (Figure 4b). In both low and mid UVB treatments, there were multiple brief phases of high reproductive output, followed by intervals of very few or no eggs being produced. *Philodina* exposed to low UVB intensity continued to reproduce up to day 73, although the mean number of offspring per female was lower in this generation. The mid UVB treatment had the highest fecundity per female, which peaked at day 25 and gradually decreased until day 61 (Figure 4).

**TABLE 4:** Cox PH regression survival analysis of maternal UVB exposure effects on offspring lifespan. Survival analyzes were done only the feeding treatments, excluding the high UVB treatment. Results examine the impact of low, mid, and high UVB treatments (1.3, 3.7, and 5.0 W/m^2^).

(a) Control versus	Coefficient ± SE	*z*	*p*
Fed control	−1.6 ± 0.5	−3.2	0.001
Fed F_1_ low	−22.6 ± 0.6	−39.0	< 0.0001
Fed F_1_ mid	−1.9 ± 0.5	−3.9	< 0.0001
Fed F_1_ high	—	—	—
Fed F_5_ low	2.5 ± 6.0	4.3	< 0.0001
Fed F_5_ mid	−18.1 ± 0.6	−31.1	< 0.0001
Fed F_5_ high	ND	ND	ND
Starved control	0.7 ± 1.0	0.73	0.4659
Starved F_1_ low	−5.7 ± 1.6	−3.50	0.0005
Starved F_1_ mid	−1.8 ± 1.2	−1.54	0.1231
Starved F_1_ high	—	—	—
Starved F_5_ low	−0.4 ± 1.2	−0.33	0.7435
Starved F_5_ mid	−3.2 ± 1.5	−2.06	0.0397
Starved F_5_ high	ND	ND	ND

(b) F_5_ versus F_1_	Estimate ± SE	Ratio	*p*
Fed control	1.6 ± 0.5	3.2	0.001
Fed low	−0.9 ± 0.8	−1.2	0.25
Mid fed	19.7 ± 0.8	25.4	< 0.0001
Fed high	ND	ND	ND
Starved control	−0.7 ± 1.0	−0.729	0.466
Starved low	−0.3 ± 0.7	−0.462	0.644
Starved mid	2.5 ± 1.2	2.049	0.040

*Note:* (a) Compares UVB treatments to generation control. (b) Estimated marginal means were used to assess the transgenerational effects by comparing F_5_ to the F_1_ generation. Coefficients are on a log scale; the Tukey method is used for comparing a family of four estimates. ND, no data (due to low reproduction of F_4_ females). Analyzes including the high UVB treatment are given in Table S3.

Maternal UVB exposure had significant effects on the net reproductive rate (*R*_0_) of bdelloid rotifers across generations. In the F_1_ generation, both fed and starved individuals exhibited reduced reproductive rates under high UVB conditions, with the lowest *R*_0_ (4.3 ± 4.8; Table 5) observed in the high UVB treatment. However, *R*_0_ was highest in the low UVB treatment (21.7 ± 11.4; Table 5). By the F_5_ generation, reproductive rates increased across treatments, with the highest *D*_0_ observed in the mid UVB group (19.1 ± 16.2; Table 5). No data were available for the high UVB treatment in F_5_ due to low reproduction among F_4_ females. Starvation reduced *R*_0_ in all groups, particularly under high UVB conditions, where reproduction was extreme low (0.1 ± 0.1 in F_1_; Table 5).

**TABLE 5:** Effects of maternal UVB exposure on net reproductive rate (*R*_0_) after parental exposure to low, mid, or high UVB radiation levels (1.3, 3.7, or 5 W/m^2^, respectively).

	Control	Low	Mid	High
F_1_ fed	9.7 ± 0.3	32.3 ± 1.1	29.5 ± 3.9	8.6 ± 1.1
F_1_ starved	4.0 ± 1.8	11.2 ± 1.3	4.9 ± 0.8	0.1 ± 0.1
F_5_ fed	26.0 ± 2.6	31.0 ± 0.9	34.4 ± 1.0	ND
F_5_ starved	11.4 ± 3.3	24.3 ± 12.3	20.5 ± 2.3	ND
Mean F_1_ net reproductive rate	6.9 ± 3.3	21.7 ± 11.4	17.2 ± 14.1	4.3 ± 4.8
Mean F_5_ net reproductive rate	13.6 ± 13.9	17.4 ± 14.4	19.1 ± 16.2	ND

*Note:* Data recorded in mean ± standard deviation.

Abbreviation: ND, no data (due to low reproduction of F_4_ females).

**FIGURE 5:**
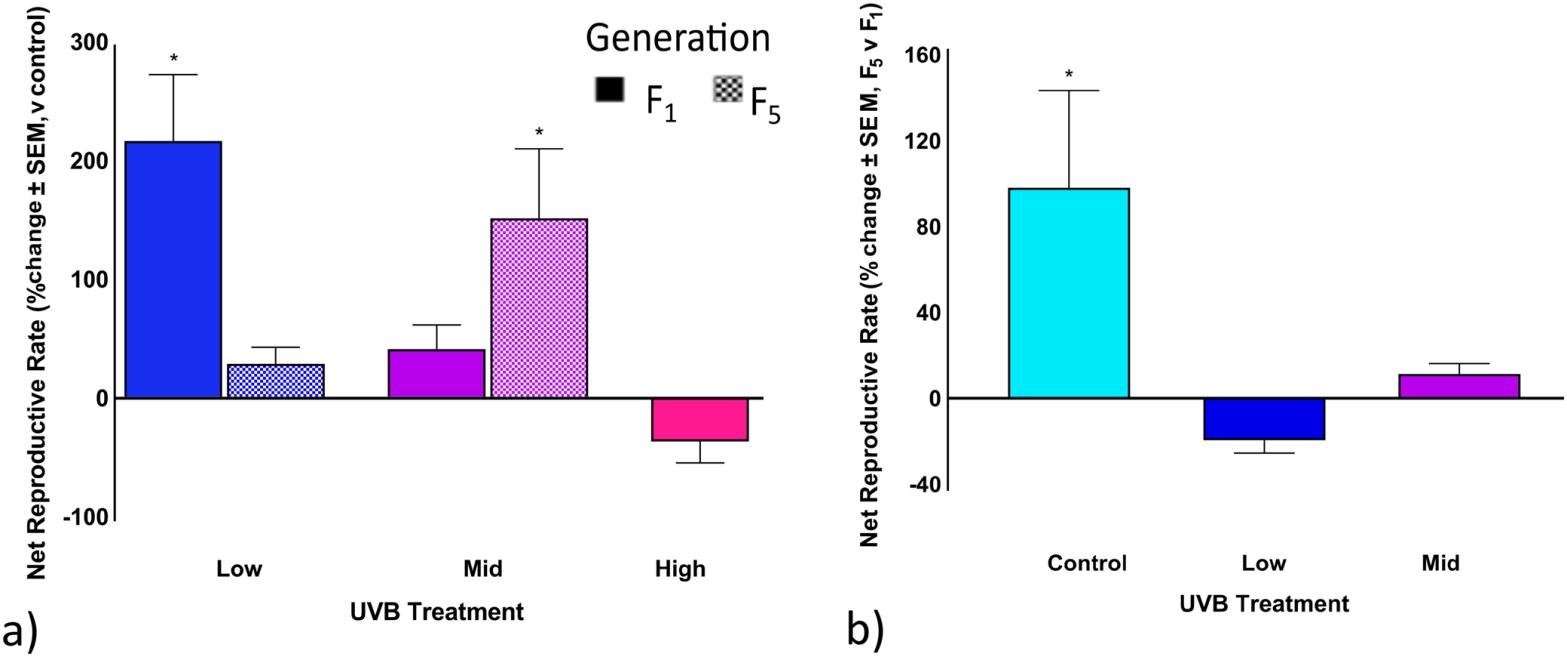
Maternal UVB exposure and transgenerational effects on *Philodina* net reproductive rate (*R*_0_). Bdelloids were exposed to three levels of UVB radiation, low, mid, and high UVB radiation (0, 1.3, 3.7, or 5.0 W/m^2^). *R*_0_ was (a) compared to the respective generation control and (b) compared for the F_5_ versus the F_1_ generation. *Estimated marginal means, *p* < 0.05. SEM, standard error of means.

When comparing the low and mid UVB treatments of the F_1_ generation to the control, *R*_0_ increased by 214.5% (Figure 5a, *p* < 0.0001; Table 6a) and 149.3%, respectively (Figure 5a, *p* = 0.001; Table 6a). Whereas high UVB exposure resulted in a 37.7% decrease (Figure 5a). In the F_5_ generation, *R*_0_ increased by 40.4% in the mid UVB treatment. The control UVB treatment showed an increase between F_1_ and F_5_ of 97.1% compared to F_1_ (Figure 5b). The mid‐UVB treatment showed an 11.1% increase (Figure 5b), while the low UVB treatment had a decline of 19.8% (Figure 5b; Table 6b).

**TABLE 6:** Analysis of UVB exposure over three generations using a linear mixed effects model (lme). *Philodina* net reproductive rate (*R*_0_) after parental exposure to low, mid, or high UVB.

(a) Control versus	Estimate ± SE	*t*	*p*
F_1_ low	−22.67 ± 2.32	−9.78	< 0.0001
F_1_ mid	−19.81 ± 2.59	−7.65	< 0.0001
F_1_ high	1.11 ± 2.32	0.43	0.97
F_5_ low	−4.96 ± 2.32	2.14	0.1751
F_5_ mid	−8.36 ± 2.32	−3.61	0.008
F_5_ high	ND	ND	ND

(b) F_5_ versus F_1_	Estimate ± SE	*t* ratio	*p*
Control	−16.33 ± 2.59	−6.31	< 0.0001
Low	1.38 ± 2.01	0.69	0.500
Mid	−4.87 ± 2.32	−2.10	0.048
High	ND	ND	ND

*Note:* (a) Compares UVB treatments to generation control. (b) Assesses the transgenerational effects by comparing F_5_ to the F_1_ generation using Kenward‐Roger correction, estimate marginal mean ± standard error (SE).

Abbreviation: ND, no data (due to low reproduction of F_4_ females).

Maternal UVB exposure influenced the generation time (T) of *Philodina* across generations, with notable variation between feeding conditions and UVB intensity. In the F_1_ generation, fed individuals exhibited the longest T under low UVB exposure (19.8 ± 3.6 days; Table 7), while the shortest occurred under high UVB (7.0 ± 3.7 days; Table 7). Starved F_1_ individuals showed less variation across UVB treatments, with slightly extended generation times compared to fed individuals except under high UVB conditions where T remained low (8.0 ± 2.8 days; Table 7). By the F_5_ generation, fed individuals showed a longer T under low and mid UVB exposure (17.3 ± 2.0 and 18.1 ± 1.3 days, respectively; Table 7). Notably, starved F_5_ individuals had drastically reduced T across treatments (1.1 ± 0.5 to 3.9 ± 2.5 days; Table 7).

**TABLE 7:** Effects of maternal UVB exposure on generation time (T, days, mean ± standard deviation) of two generations (F_1_ and F_5_) of *Philodina* after maternal exposure to low, mid, high, and control UVB radiation (0, 1.3, 3.7, or 5.0 W/m^2^).

	Control	Low	Mid	High
F_1_ fed	9.7 ± 0.5	19.8 ± 3.6	16.4 ± 1.5	7.0 ± 3.7
F_1_ starved	12.6 ± 5.2	15.6 ± 1.9	16.2 ± 0.8	8.0 ± 2.8
F_5_ fed	12.3 ± 2.1	17.3 ± 2.0	18.1 ± 1.3	ND
F_5_ starved	1.1 ± 0.5	3.9 ± 2.5	3.9 ± 2.5	ND
Mean F_1_ generation time	11.2 ± 3.7	17.7 ± 2.9	16.3 ± 1.1	7.4 ± 3.0
Mean F_5_ generation time	11.8 ± 2.5	20.8 ± 10.8	19.4 ± 2.3	ND

Abbreviation: ND, no data (due to low reproduction of F_4_ females).

**FIGURE 6:**
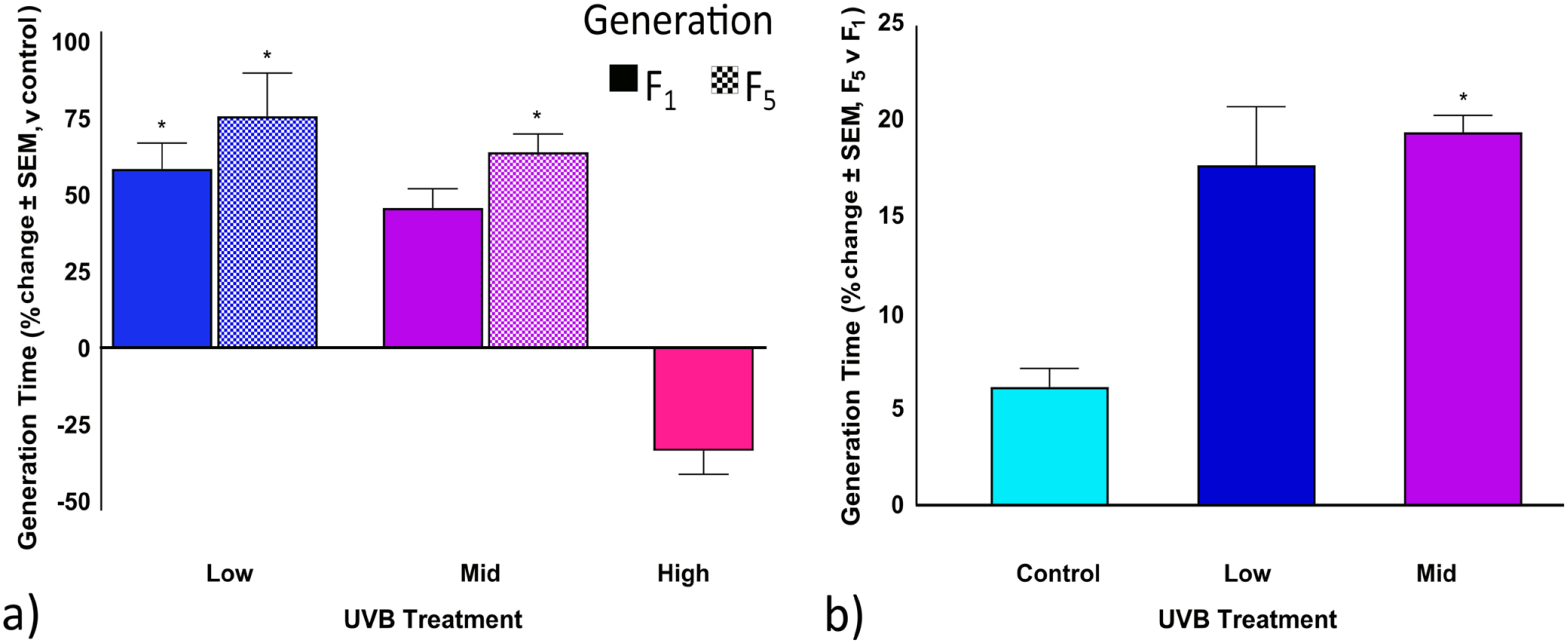
Maternal UVB exposure and transgenerational effects on *Philodina* generation time. Bdelloids were exposed to three levels of UVB radiation (0, 1.3, 3.7, or 5.0 W/m^2^). Life history traits were (a) compared to the respective generational control and (b) compared for the F_5_ versus the F_1_ generation. *Estimated marginal means, *p* < 0.05. SEM, standard error of means.

In the F_1_ generation, low and mid UVB treatments resulted in a 58.0% and 45.5% increase (Figure 5a), respectively, in generation time when compared to the control, whereas high UVB exposure decreased generation time by 33.9% (Figure 6a, *p* = 0.0006; Table 8a). In the F_5_ generation, generation time increased by 76.3% in the low UVB treatments (Figure 6a; *p* = 0.0305; Table S4) and by 64.4% in the mid UVB treatments (Figure 6a; *p* < 0.0001; Table 8a) relative to the F_5_ control (Figure 6a; *p* < 0.0001; Table 8a). An increase of 19.0% for the mid UVB treatment (Figure 6b; *p* < 0.0001; Table 8b) was observed, though none were significant. An increase in T was also observed in the low UVB treatment; however, pairwise comparisons revealed that the difference was not statistically significant. A comparison of estimated marginal means analysis resulted in significant interaction in the low F_5_ generations that was not detected by the Cox PH model; see Table S4.

**TABLE 8:** Cox PH regression analysis of maternal UVB exposure effects on offspring generation time. Survival analysis of the effects of maternal UVB exposure at low, mid, high UVB treatments (1.3, 3.7, 5.0 W/m^2^) on offspring generation time (*T*).

(a) control versus	Coefficient ± SE	*z*	*p*
Generations (F_1_ vs. F_5_)	0.7 ± 0.7	0.980	0.327
Starved versus fed	−0.3 ± 0.3	−0.884	0.377
F_1_ low	−1.6 ± 0.6	−2.748	0.006
F_1_ mid	−0.7 ± 0.6	−1.077	0.281
F_1_ high	2.0 ± 0.7	2.756	0.006
F_5_ low^a^	−1.2 ± 0.9	−1.362	0.173
F_5_ mid	−1.9 ± 0.9	−2.066	0.039
F_5_ high	ND	ND	ND

(b) F_5_ versus F_1_	Estimate ± SE	*z* ratio	*p*
Control	−0.7 ± 0.7	−0.980	0.3272
Low	0.5 ± 0.5	1.018	0.3087
Mid	1.3 ± 0.6	2.238	0.0250
High	ND	ND	ND

*Note:* (a) Compares UVB treatments to generation control. (b) Estimated marginal means were used to assess the transgenerational effects by comparing F_5_ to the F_1_ generation. Coefficients are log scale; Tukey method for comparing a family of 4 estimates.

Abbreviation: ND, no data (due to low reproduction of F_4_ females).

^a^ Comparison of estimated marginal means analysis resulted in significant interaction that was not detected by the Cox PH model, see Table S4.

**FIGURE 7:**
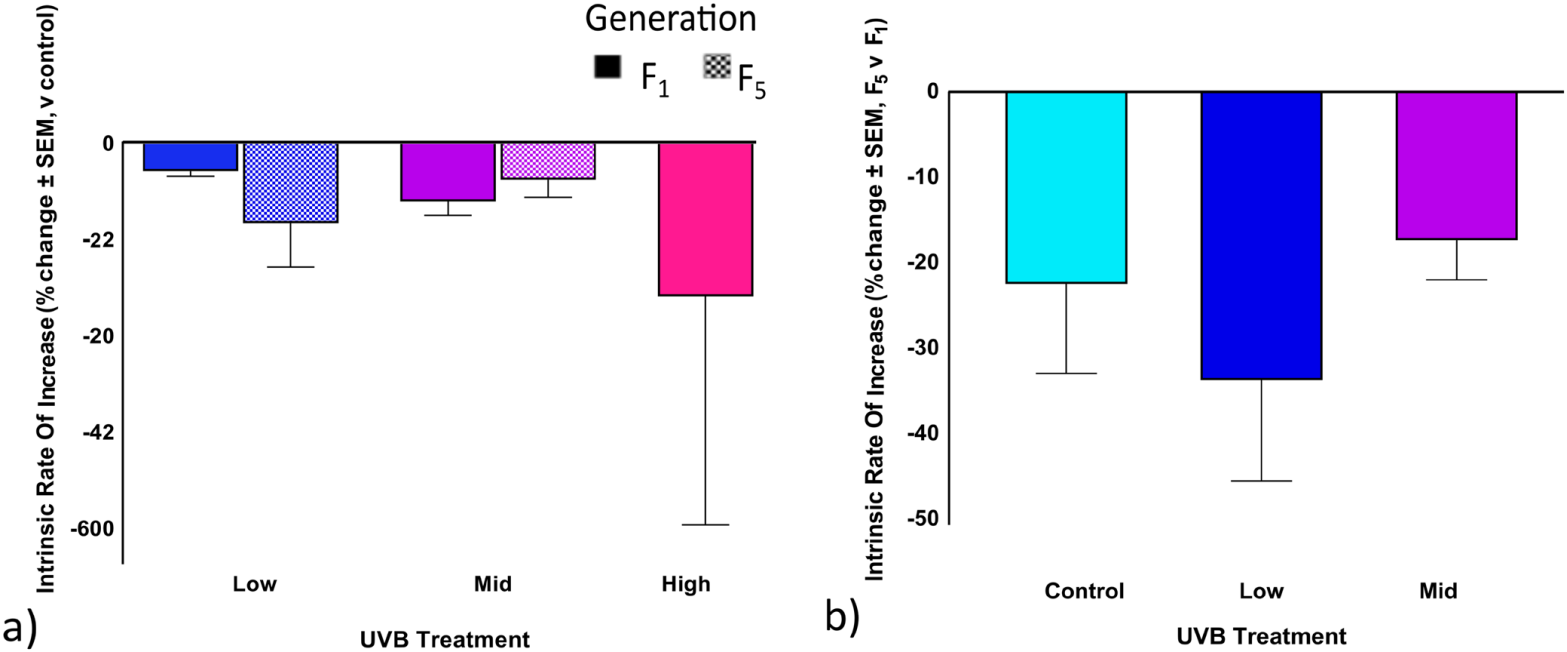
Maternal UVB exposure and transgenerational effects on *Philodina* intrinsic rate of change. Bdelloids were exposed to three levels of UVB radiation: Low, mid, and high (0, 1.3, 3.7, or 5.0 W/m^2^). Life history traits were (a) compared to the respective generational control and (b) compared for the F_5_ versus the F_1_ generation.

Maternal UVB exposure affected the intrinsic rate of increase (*r*) in *Philodina*, with differences observed across UVB intensities, generations, and feeding conditions. In the F_1_ generation, fed individuals showed relatively stable *r* values across treatments, ranging from 0.35 ± 0.08 for the high to 0.18 ± 0.01 for low UV treatment; however, in the starved F_1_ individuals *r* decreased to −0.26 ± 0.37 in the high UV treatment (Table 9). By the F_5_ generation, fed individuals maintained moderate *r* values under low and mid‐UVB conditions (0.20 ± 0.01 for both; Table 9). Starved F_5_ individuals exhibited near‐zero *r* values across all treatments. When comparing UVB treatments to their generation controls, no differences were detected among the UVB treatments in either the F_1_ or F_5_ generations (Figure 7a). When comparing F_5_ to F_1_, again none of the changes observed were significant (Table 10a,b).

**TABLE 9:** Effects of UVB exposure on intrinsic rate of increase (*r*, days means ± standard deviation) after maternal exposure to low, mid, or high UVB radiation levels (1.3, 3.7, or 5 W/m^2^, respectively).

	Control	Low	Mid	High
F_1_ Fed	0.23 ± 0.01	0.18 ± 0.01	0.21 ± 0.01	0.35 ± 0.08
F_1_ Starved	0.15 ± 0.08	0.14 ± 0.02	0.10 ± 0.01	−0.26 ± 0.37
F_5_ Fed	0.27 ± 0.02	0.20 ± 0.01	0.20 ± 0.01	ND
F_5_ Starved	0.01 ± 0.05	0.02 ± 0.11	0.05 ± 0.06	ND
Mean F_1_ intrinsic rate of increase	0.18 ± 0.08	0.17 ± 0.02	0.15 ± 0.06	0.11 ± 0.36
Mean F_5_ intrinsic rate of increase	0.14 ± 0.15	0.11 ± 0.12	0.13 ± 0.08	ND

Abbreviation: ND, no data (due to low reproduction of F_4_ females).

**TABLE 10:** Analysis of UVB exposure over three generations using a linear mixed effects model (lme). *Philodina* intrinsic rate of increases (*r*) of change after parental exposure to low, mid, or high UVB.

(a) Control versus	Estimate ± SE	*t*	*p*
F_1_ Low	0.013 ± 0.52	0.25	0.99
F_1_ Mid	0.027 ± 0.06	0.47	0.97
F_1_ High	0.089 ± 0.05	1.45	0.49
F_5_ Low	0.029 ± 0.05	0.55	0.94
F_5_ Mid	0.013 ± 0.05	0.26	0.99
F_5_ High	ND	ND	ND

(b) F_5_ versus F_1_	Estimate ± SE	*t* ratio	*p*
Control	0.04 ± 0.06	0.70	0.49
Low	0.06 ± 0.05	1.25	0.23
Mid	0.03 ± 0.05	0.51	0.62
High	ND	ND	ND

*Note:* (a) Compares UVB treatments to generation control. (b) Assesses the transgenerational effects by comparing the F_5_ to the F_1_ generation using Kenward‐Roger correction, estimate ± standard error (SE).

Abbreviation: ND, no data (due to low reproduction of F_4_ females).

## 5 Discussion

Despite extensive research on UVR tolerance in aquatic organisms, the effects of maternal UVB exposure on rotifer life history traits across multiple generations remain poorly understood. Our study highlights the critical role of UVB as an environmental stressor that influences lifespan, reproductive success, and transgenerational adaptation in bdelloid rotifers. Our findings showed that low and mid UVB exposure led to extended lifespans and enhanced reproduction over generations in a desert rock pool bdelloid from desert rock pools. Winter UVB intensities at Hueco Tanks align with our low UVB treatment, reinforcing the ecological relevance of our findings. Under high temperatures and rapid water evaporation, bdelloids may prioritize reproduction at the cost of lifespan to maximize reproductive success within a shortened hydroperiod, as was seen in the mid UVB treatment.

Bdelloid rotifers exhibit a wide range of life history traits, with lifespan, reproduction, and generation time varying across species and environmental conditions (King et al. 2005). Our lifetable analyzes showed that *Philodina* exposed to low and mid UVB treatments had lifespan and reproductive rates within the upper range reported for bdelloid species. Under optimal conditions, bdelloids live around 35 days (e.g., *Philodina vorax* (Janson, 1893): 22 days (Ricci and Fascio 1995); *P. roseola* : 48 days (Ricci 1983); *Philodina gregaria* Murray, 1910: 89 days (Dartnall 1992)). In addition, fecundity under low and mid UVB exposure treatments for *Philodina* was comparable to the highest recorded values for bdelloids ( *P. roseola* , 30 offspring; Ricci 1983). However, despite high reproductive rates, the generation time of *Philodina* was longer under low and mid UVB treatments than typical for bdelloids (King et al. 2005). In contrast, exposure to high UVB intensity or no exposure resulted in reduced fecundity and no significant reproductive advantage, suggesting that both extremes may be detrimental to reproductive success. These results indicate that bdelloid rotifers have adapted to regional UVR levels, where moderate UV exposure promotes survival and reproductive success, while extreme UV conditions may trigger maladaptive stress responses.

The trade‐off between somatic maintenance and reproduction is a common response to environmental stressors, including UVB radiation (Heine et al. 2019; Kan et al. 2023; Sha et al. 2020). In rotifers, moderate UVB exposure may also promote adaptive maternal effects enhancing both lifespan and reproductive output, while extreme UVB exposure results in reduced fitness (Heine et al. 2019; Fischer et al. 2013; Kan et al. 2023; Zhu et al. 2021). For example, in *Brachionus asplanchnoidis* Müller 1863, low UVB exposure promoted survival, but higher intensities reduced longevity and reproduction (Kan et al. 2023). Similarly, *R. rotatoria* lifespan decreased from 27 days under normal conditions to 8 days under prolonged low UV exposure (Zhu et al. 2021). These shifts of resource allocation to repair and maintenance and reduced reproductive output have also been observed in daphnids (Oexle et al. 2016), copepods (Heine et al. 2019; Hylander et al. 2013; Moeller et al. 2005), and tardigrades (Altiero et al. 2010). These results demonstrate patterns where moderate UVB exposure enhances survival or reproduction, but prolonged or intense UVB exposure diverts resources toward cellular maintenance.

Despite the potential for adaptive maternal effects, our findings reveal the negative survival impacts of multi‐generational UVB exposure. When maternal lines of *Philodina* were exposed to UVB for a single generation, survival rates remained high across all UVB intensities. However, repeated exposure led to a progressive decline in survival across generations. This transgenerational damage could occur due to epigenetic modifications, altered gene expression, or altered germ cell integrity (Dam 2013; Fox et al. 2019; White and Butlin 2021). While our results suggest that maternal effects may offer some initial protection against UVB stress, they do not appear to mitigate the long‐term consequences of repeated exposure.

One potential factor contributing to the increasing vulnerability of *Philodina* to UVB exposure is the loss of red pigmentation after laboratory culturing (Baeza and Walsh 2024). These bdelloids were initially brightly pigmented, likely due to carotenoids obtained from their diet, which may provide antioxidant protection against UVR damage (Fischer et al. 2013; Snare et al. 2013). We observed that eggs laid by the parental generation remained pigmented even after several days in the laboratory without access to dietary pigments. This suggests a form of maternal investment, where females transfer carotenoids or other protective compounds to their offspring, providing temporary UVR protection during early development. A similar strategy is observed in the copepod *Leptodiaptomus minutus* (Lilljeborg in Guerne & Richard, 1889) where females allocated carotenoids and fatty acids to their eggs to enhance offspring survival under intense UVR conditions (Schneider et al. 2016). Similarly, in *A. tonsa* , when maintained on a diet rich in photoprotective compounds, had increased survival to UVB exposure (Hylander et al. 2013). Pigmentation in *Philodina* might indicate an adaptive response to moderate UVR environments that ensure survival. Maternal provisioning and the resulting pigmentation enable offspring to rapidly adjust their phenotype, allowing them to respond quickly to environmental stressors.

Our results suggest that moderate UVB stress may optimize reproductive success through trade‐offs between longevity and fecundity, whereas both extreme UVB exposure and its absence negatively impact these traits. This pattern indicates that *Philodina* may have locally adapted to regional UVB levels. Seasonal fluctuations in UVB intensity and temperature in the Chihuahuan Desert drive these adaptive responses, as bdelloids must balance survival strategies with reproductive investment in ephemeral habitats. The capacity to maintain reproductive success and longevity under moderate UVB conditions may be crucial for bdelloid populations facing increasingly variable climate conditions. As climate change continues to alter UVB exposure and environmental stability, understanding bdelloid life history traits will be essential for predicting their future population resilience.

## 6 Future Directions

Our study provides evidence supporting regional adaptation to UVB intensity in bdelloid rotifers, but further research is needed to elucidate the underlying mechanisms. One key limitation is the uncertainty regarding whether the observed responses result from genetic adaptation or maternal effects. To better understand bdelloid responses to UVR, future studies should quantify gene expression changes or track changes in allele frequency in rotifers exposed to environmentally relevant levels of UVB across multiple generations. Tests evaluating antioxidant capacity or DNA repair mechanisms could distinguish between genotypic and phenotypic responses. Additionally, identifying the specific pigments involved and their sources would be valuable for maintaining pigmentation in laboratory settings and assessing its functional role in UV protection. Experiments incorporating a broader range of environmental factors, such as temperature fluctuations and photoperiod variation, could provide deeper insight into the adaptive strategies employed by aquatic invertebrates in shallow waters.

## Author Contributions

**Maribel J. Baeza:** conceptualization (equal), data curation (equal), formal analysis (equal), funding acquisition (supporting), investigation (equal), methodology (equal), project administration (supporting), resources (supporting), software (equal), supervision (supporting), validation (equal), visualization (lead), writing – original draft (lead), writing – review and editing (equal). **Elizabeth J. Walsh:** conceptualization (equal), data curation (equal), formal analysis (equal), funding acquisition (lead), investigation (equal), methodology (equal), project administration (lead), resources (lead), software (equal), supervision (lead), validation (equal), visualization (supporting), writing – original draft (supporting), writing – review and editing (equal).

## Disclosure

Any opinions, findings, conclusions, or recommendations expressed in this material are those of the author(s) and do not necessarily reflect the views of the National Science Foundation.

## Conflicts of Interest

The authors declare no conflicts of interest.

## Supporting information


**Table S1:** Comparison of pigmentation levels between generations. Results of a one‐way ANOVA (pigment ~ Gen, data = pigm.data).
**Table S2:** Pairwise comparison of survival of *Philodina* post exposure to 0, 1.3, 3.7, or 5.0 W/m^2^ of UVB intensity, of three non‐consecutive generations (F_0_, F_2_, F_4_) using Tukey's tests, after a two‐way ANOVA.
**Table S3:** Cox proportional hazard regression including the high UVB treatment. Survival analysis of the effects of maternal UVB exposure at low, mid, high UVB treatments (1.3, 3.7, 5.0 W/m^2^) on lifespan. Degrees of freedom method: Kenward‐Roger comparing a family of four estimates. ND, no data (due to low reproduction of F_4_ females).
**Table S4:** Comparison of estimated marginal means used as a post hoc analysis to verify interaction detected using the Cox proportional hazard regression model, using Tukey adjustments. emmeans(mod.T.0,pairwise~UV|Gen,adsjust = “Tukey”).

## Data Availability

The data that support the findings of this study are openly available in UTEP Bioinformatics Data Repository at https://datarepo.bioinformatics.utep.edu/getdata?acc=PGPYEYVM7P5V62R.
