# Supplementary material for: Local Adaptation? Enhanced Fitness Under Regional UVB Intensities in a Rock Pool Bdelloid Rotifer
Source: Ecol Evol. 2025 Oct 28;15(10):e72256. doi: 10.1002/ece3.72256 (PMC12559811; doi:10.1002/ece3.72256)
Supplement: Supplementary file 1 — Table S1: Comparison of pigmentation levels between generations. Results of a one‐way ANOVA (pigment ~ Gen, data = pigm.data). Table S2: Pairwise comparison of survival of Philodina post exposure to 0, 1.3, 3.7, or 5.0 W/m2 of UVB intensity, of three non‐consecutive generations (F0, F2, F4) using Tukey's tests, after a two‐way ANOVA. Table S3: Cox proportional hazard regression including the high UVB treatment. Survival analysis of the effects of maternal UVB exposure at low, mid, high UVB treatments (1.3, 3.7, 5.0 W/m2) on lifespan. Degrees of freedom method: Kenward‐Roger comparing a family of four estimates. ND, no data (due to low reproduction of F4 females). Table S4: Comparison of estimated marginal means used as a post hoc analysis to verify interaction detected using the Cox proportional hazard regression model, using Tukey adjustments. emmeans(mod.T.0,pairwise~UV|Gen,adsjust = “Tukey”). [file ECE3-15-e72256-s001.docx]

**Appendix**

**Pigment Analysis**

**Table S1.** Comparison of pigmentation levels between generations. a) Results of a One-Way ANOVA (pigment ~ Gen, data = pigm.data).

Please check

| a) | diff | p value |
| --- | --- | --- |
| Generation | 2 | <0.0001 |
| F_2_-F_0_ | 91.2 | 0.008 |
| F_4_-F_2_ | 200.5 | 0 |
| F_4_-F_2_ | 109.3 | 0.001 |

**Generational UVR exposure**

Survival was analyzed in the F_0_, F_2_, and F_4_ generations. Each UVB treatment was maintained continuously and exposed when at least 800 individuals were 10 days or older. To evaluate the effects of UVB intensity on survival, a Two-Way analysis of variance (ANOVA) (Table 2) was performed to compare effects of UVB treatments and generation (aov(recovered ~ uvb*gen)). When significant differences were detected, a Tukey post hoc test was conducted to determine pairwise differences between UVB treatment groups. Statistical analyses were performed using R version 3.4.3 (R Core Team, 2022) and RStudio version 1.0.136 (RStudio Team, 2020).

**Table S2.** Pairwise comparison of survival of *Philodina* post exposure to 0, 1.3, 3.7, or 5.0 W/m^2^ of UVB intensity, of three non-consecutive generations (F_0_, F_2_, F_4_) using Tukey tests, after a Two-way ANOVA.

| **a.** | **Generation** | **diff** | **lwr** | **upr** | **p adj** |
| --- | --- | --- | --- | --- | --- |
|  | F_2_-F_0_ | -0.2098 | -0.26331 | -0.15629 | 0.0000000 |
|  | F_4_-F_0_ | -0.29125 | -0.34476 | -0.23773 | 0.0000000 |
|  | F_4_-F_2_ | -0.08145 | -0.13496 | -0.02794 | 0.0012047 |
| **b.** | **UVB** | **diff** | **lwr** | **upr** | **p adj** |
|  | 130-0 | -0.2352 | -0.303 | -0.16739 | 0.00e+00 |
|  | 370-0 | -0.35435 | -0.42215 | -0.28654 | 0.00e+00 |
|  | 500-0 | -0.6425 | -0.71031 | -0.5747 | 0.00e+00 |
|  | 370-130 | -0.11915 | -0.18695 | -0.05135 | 5.62e-05 |
|  | 500-130 | -0.40731 | -0.47511 | -0.3395 | 0.00e+00 |
|  | 500-370 | -0.28816 | -0.35596 | -0.22035 | 0.00e+00 |
| **c.** | **Generation: UVB** | **diff** | **lwr** | **upr** | **p adj** |
|  | F_2_:0-F_0_:0 | -0.15957 | -0.30954 | -0.00959 | 0.0261867 |
|  | F_4_:0-F_0_:0 | -0.19805 | -0.34802 | -0.04807 | 0.0012210 |
|  | F_0_:130-F_0_:0 | -0.21399 | -0.36397 | -0.06402 | 0.0002837 |
|  | F_2_:130-F_0_:0 | 0.019927 | -0.13005 | 0.169903 | 0.9999993 |
|  | F_4_:130-F_0_:0 | -0.21899 | -0.36897 | -0.06902 | 0.0001759 |
|  | F_0_:370-F_0_:0 | -0.31649 | -0.46647 | -0.16652 | 0.0000000 |
|  | F_2_:370-F_0_:0 | -0.89524 | -1.04522 | -0.74527 | 0.0000000 |
|  | F_4_:370-F_0_:0 | -0.02564 | -0.17562 | 0.124333 | 0.9999902 |
|  | F_0_:500-F_0_:0 | -0.33274 | -0.48272 | -0.18277 | 0.0000000 |
|  | F_2_:500-F_0:_0 | -0.55422 | -0.70419 | -0.40424 | 0.0000000 |
|  | F_4_:500-F_0_:0 | -0.82399 | -0.97397 | -0.67402 | 0.0000000 |
|  | F_4_:0-F_2_:0 | -0.03848 | -0.18845 | 0.111496 | 0.9994505 |
|  | F_0_:130-F_2_:0 | -0.05443 | -0.2044 | 0.095549 | 0.9882566 |
|  | F_2_:130-F_2_:0 | 0.179494 | 0.029518 | 0.329469 | 0.0058410 |
|  | F_4_:130-F_2_:0 | -0.05943 | -0.2094 | 0.090549 | 0.9766052 |
|  | F_0_:370-F_2_:0 | -0.15693 | -0.3069 | -0.00695 | 0.0314528 |
|  | F_2_:370-F_2_:0 | -0.73568 | -0.88565 | -0.5857 | 0.0000000 |
|  | F_4_:370-F_2_:0 | 0.133924 | -0.01605 | 0.283899 | 0.1304286 |
|  | F_0_:500-F_2_:0 | -0.17318 | -0.32315 | -0.0232 | 0.0096034 |
|  | F_2_:500-F_2_:0 | -0.39465 | -0.54462 | -0.24467 | 0.0000000 |
|  | F_4_:500-F_2_:0 | -0.66443 | -0.8144 | -0.51445 | 0.0000000 |
|  | F_0_:130-F_4_:0 | -0.01595 | -0.16592 | 0.134029 | 0.9999999 |
|  | F_2_:130-F_4_:0 | 0.217973 | 0.067998 | 0.367949 | 0.0001941 |
|  | F_4_:130-F_4_:0 | -0.02095 | -0.17092 | 0.129029 | 0.9999988 |
|  | F_0_:370-F_4_:0 | -0.11845 | -0.26842 | 0.031529 | 0.2791176 |
|  | F_2_:370-F_4_:0 | -0.6972 | -0.84717 | -0.54722 | 0.0000000 |
|  | F_4_:370-F_4_:0 | 0.172403 | 0.022428 | 0.322379 | 0.0101920 |
|  | F_0_:500-F_4_:0 | -0.1347 | -0.28467 | 0.015279 | 0.1250187 |
|  | F_2_:500-F_4_:0 | -0.35617 | -0.50615 | -0.20619 | 0.0000000 |
|  | F_4_:500-F_4_:0 | -0.62595 | -0.77592 | -0.47597 | 0.0000000 |
|  | F_2_:130-F_0_:130 | 0.23392 | 0.083944 | 0.383895 | 0.0000401 |
|  | F_4_:130-F_0_:130 | -0.005 | -0.15498 | 0.144975 | 1.0000000 |
|  | F_0_:370-F_0_:130 | -0.1025 | -0.25248 | 0.047475 | 0.5065167 |
|  | F_2_:370-F_0_:130 | -0.68125 | -0.83123 | -0.53127 | 0.0000000 |
|  | F_4_:370-F_0_:130 | 0.18835 | 0.038374 | 0.338325 | 0.0028197 |
|  | F_0_:500-F_0_:130 | -0.11875 | -0.26873 | 0.031225 | 0.2754436 |
|  | F_2_:500-F_0_:130 | -0.34022 | -0.4902 | -0.19025 | 0.0000000 |
|  | F_4_:500-F_0_:130 | -0.61 | -0.75998 | -0.46002 | 0.0000000 |
|  | F_4_:130-F_2_:130 | -0.23892 | -0.3889 | -0.08894 | 0.0000240 |
|  | F_0_:370-F_2_:130 | -0.33642 | -0.4864 | -0.18644 | 0.0000000 |
|  | F_2_:370-F_2_:130 | -0.91517 | -1.06515 | -0.76519 | 0.0000000 |
|  | F_4_:370-F_2_:130 | -0.04557 | -0.19555 | 0.104405 | 0.9974040 |
|  | F_0_:500-F_2_:130 | -0.35267 | -0.50265 | -0.20269 | 0.0000000 |
|  | F_2_:500-F_2_:130 | -0.57414 | -0.72412 | -0.42417 | 0.0000000 |
|  | F_4_:500-F_2_:130 | -0.84392 | -0.9939 | -0.69394 | 0.0000000 |
|  | F_0_:370-F_4_:130 | -0.0975 | -0.24748 | 0.052475 | 0.5856934 |
|  | F_2_:370-F_4_:130 | -0.67625 | -0.82623 | -0.52627 | 0.0000000 |
|  | F_4_:370-F_4_:130 | 0.19335 | 0.043374 | 0.343325 | 0.0018405 |
|  | F_0_:500-F_4_:130 | -0.11375 | -0.26373 | 0.036225 | 0.3395911 |
|  | F_2_:500-F_4_:130 | -0.33522 | -0.4852 | -0.18525 | 0.0000000 |
|  | F_4_:500-F_4_:130 | -0.605 | -0.75498 | -0.45502 | 0.0000000 |
|  | F_2_:370-F_0_:370 | -0.57875 | -0.72873 | -0.42877 | 0.0000000 |
|  | F_4_:370-F_0_:370 | 0.29085 | 0.140874 | 0.440825 | 0.0000001 |
|  | F_0_:500-F_0_:370 | -0.01625 | -0.16623 | 0.133725 | 0.9999999 |
|  | F_2_:500-F_0_:370 | -0.23772 | -0.3877 | -0.08775 | 0.0000272 |
|  | F_4_:500-F_0_:370 | -0.5075 | -0.65748 | -0.35752 | 0.0000000 |
|  | F_4_:370-F_2_:370 | 0.8696 | 0.719624 | 1.019575 | 0.0000000 |
|  | F_0_:500-F_2_:370 | 0.5625 | 0.412525 | 0.712475 | 0.0000000 |
|  | F_2_:500-F_2_:370 | 0.341027 | 0.191051 | 0.491002 | 0.0000000 |
|  | F_4_:500-F_2_:370 | 0.07125 | -0.07873 | 0.221225 | 0.9164575 |
|  | F_0_:500-F_4_:370 | -0.3071 | -0.45708 | -0.15712 | 0.0000000 |
|  | F_2_:500-F_4_:370 | -0.52857 | -0.67855 | -0.3786 | 0.0000000 |
|  | F_4_:500-F_4_:370 | -0.79835 | -0.94833 | -0.64837 | 0.0000000 |
|  | F_2_:500-F_0_:500 | -0.22147 | -0.37145 | -0.0715 | 0.0001383 |
|  | F_4_:500-F_0_:500 | -0.49125 | -0.64123 | -0.34127 | 0.0000000 |
|  | F_4_:500-F_2_:500 | -0.26978 | -0.41975 | -0.1198 | 0.0000009 |

**Life history characteristics**

**Lifespan**

Life history parameters were analyzed to assess the effects of maternal UVB exposure on offspring fitness. Multivariate Cox proportional hazards (PH) regression survival analysis (Kragh Andersen et al. 2021) were used to evaluate the impact of single or multiple maternal UVB exposures on lifespan and generation time (T) in the F_1_and F_5_ generations. Cox PH models were fitted using the Survival package (version 3.3-1; Therneau 2022), and mixed-effects Cox PH models were implemented with the coxme package (version 2.2-18.1; Therneau & Grambsch 2000A, B). Maternal UVB exposure had variable effects on the lifespan of *Philodina* across generations, feeding conditions (fed versus starved), and UVB intensity. Lifespan was shortest under high UVB exposure with no significant effect found in the Cox PH model when the high UVB treatment was included in the lifespan analysis.

**Table S3**. Cox proportional hazard regression including the high UVB treatment. Survival analysis of the effects of maternal UVB exposure at low, mid, high UVB treatments (1.3, 3.7, 5.0 W/m^2^) on lifespan. Degrees-of-freedom method: Kenward-Roger comparing a family of 4 estimates. ND= No Data (due to low reproduction of F_4_ females).

| **a)** | | coefficient ± SE | z value | Pr(>\|z\|) | |
| --- | --- | --- | --- | --- | --- |
| Generation Intersect | -1.116 ± 0.70 | | -1.586 | 0.113 |  |
| Fed Intersect | 1.757 ± 0.31 | | 4.918 | <0.001 |  |
| F_1_ low | -5 .295 ± 0.88 | | -6.002 | <0.001 |  |
| F_1_ mid | -1.963 ± 0.69 | | -2.836 | 0.0046 |  |
| F_1_ high | 0.927 ± 0.63 | | 1.488 | 0.1366 |  |
| F_5_ low | -1.1743± 0.85 | | 2.040 | 0.0414 |  |
| F_5_ mid | -1.103 ± 0.89 | | -1.245 | 0.2132 |  |
| F_5_ high | 21.54 ± 5626 | | 0.004 | 0.9969 |  |
| **b)** | **Estimate ± SE** | | **z ratio** | **pvalue** |  |
| Control | 1.116 ± 0.70 | | 1.586 | 0.1128 |  |
| Low | -0.626 ± 0.49 | | -1.282 | 0.1997 |  |
| Mid | 2.219 ± 0.65 | | 3.418 | 0.0006 |  |
| High | -20.428 ± 5626.1 | | -0.004 | 0.997 |  |

**Generation Time**

Estimated Marginal Means were calculated using the lme4 package (Bates et al. 2015) to compare lifespan differences between treatments as well as F_5_ and F_1_ across UVB exposure levels. All statistical analyses were conducted in R (version 3.4.3; R Core Team 2022) and RStudio (version 1.0.136). comparison of estimated marginal means analysis resulted in significant interaction that was not detected by the Cox proportional model see Appendix Table S3.

**Table S4**. Comparison of estimated marginal means used as a post-hoc analysis to verify interaction detected using the Cox proportional hazard regression model, using Tukey adjustments. emmeans(mod.T.0,pairwise~UV|Gen,adsjust="Tukey")

| **a)** | | coefficient ± SE | z value | Pr(>\|z\|) | |
| --- | --- | --- | --- | --- | --- |
| ed Intersect | 1.616 ± 0.59 | | 2.748 | 0.0305 |  |
| F_1_ low | 1.757 ± 0.31 | | 4.918 | <0.001 |  |
| F_1_ mid | 0.671 ± 0.62 | | 1.077 | 0.704 |  |
| F_1_ high | -1.956 ± 0.71 | | -2.756 | 0.030 |  |
| F_5_ low | 2.814 ± 0.69 | | 4.098 | 0.0002 |  |
| F_5_ mid | 2.583 ± 0.64 | | 4.014 | 0.0003 |  |
| F_5_ high | -4.380 ± 1.20 | | -3.656 | 0.0015 |  |
| 1. **F_1_ v F_5_** | **Estimate ± SE** | | **z ratio** | **pvalue** |  |
| Control | ± | |  |  |  |
| Low | 2.814 ± 0.69 | | 4.098 | 0.0002 |  |
| Mid | 2.583 ± 0.64 | | 4.014 | 0.0003 |  |
| High | -4.380 ± 1.99 | | -3.656 | 0.0015 |  |
